# Machine learning algorithms to predict healthcare-seeking behaviors of mothers for acute respiratory infections and their determinants among children under five in sub-Saharan Africa

**DOI:** 10.3389/fpubh.2024.1362392

**Published:** 2024-06-19

**Authors:** Tirualem Zeleke Yehuala, Muluken Chanie Agimas, Nebiyu Mekonnen Derseh, Sisay Maru Wubante, Bezawit Melak Fente, Getaneh Awoke Yismaw, Tigabu Kidie Tesfie

**Affiliations:** ^1^Department Health Informatics, Institute of Public Health, College of Medicine and Health Sciences, University of Gondar, Gondar, Ethiopia; ^2^Department of Epidemiology and Biostatistics, Institute of Public Health, College of Medicine and Health Sciences, University of Gondar, Gondar, Ethiopia; ^3^Department of General Midwifery, School of Midwifery, College of Medicine and Health Sciences, University of Gondar, Gondar, Ethiopia

**Keywords:** acute respiratory infections, health-seeking behaviors, prediction, machine learning algorithms, sub-Saharan Africa

## Abstract

**Background:**

Acute respiratory infections (ARIs) are the leading cause of death in children under the age of 5 globally. Maternal healthcare-seeking behavior may help minimize mortality associated with ARIs since they make decisions about the kind and frequency of healthcare services for their children. Therefore, this study aimed to predict the absence of maternal healthcare-seeking behavior and identify its associated factors among children under the age 5 in sub-Saharan Africa (SSA) using machine learning models.

**Methods:**

The sub-Saharan African countries’ demographic health survey was the source of the dataset. We used a weighted sample of 16,832 under-five children in this study. The data were processed using Python (version 3.9), and machine learning models such as extreme gradient boosting (XGB), random forest, decision tree, logistic regression, and Naïve Bayes were applied. In this study, we used evaluation metrics, including the AUC ROC curve, accuracy, precision, recall, and F-measure, to assess the performance of the predictive models.

**Result:**

In this study, a weighted sample of 16,832 under-five children was used in the final analysis. Among the proposed machine learning models, the random forest (RF) was the best-predicted model with an accuracy of 88.89%, a precision of 89.5%, an F-measure of 83%, an AUC ROC curve of 95.8%, and a recall of 77.6% in predicting the absence of mothers’ healthcare-seeking behavior for ARIs. The accuracy for Naïve Bayes was the lowest (66.41%) when compared to other proposed models. No media exposure, living in rural areas, not breastfeeding, poor wealth status, home delivery, no ANC visit, no maternal education, mothers’ age group of 35–49 years, and distance to health facilities were significant predictors for the absence of mothers’ healthcare-seeking behaviors for ARIs. On the other hand, undernourished children with stunting, underweight, and wasting status, diarrhea, birth size, married women, being a male or female sex child, and having a maternal occupation were significantly associated with good maternal healthcare-seeking behaviors for ARIs among under-five children.

**Conclusion:**

The RF model provides greater predictive power for estimating mothers’ healthcare-seeking behaviors based on ARI risk factors. Machine learning could help achieve early prediction and intervention in children with high-risk ARIs. This leads to a recommendation for policy direction to reduce child mortality due to ARIs in sub-Saharan countries.

## Introduction

In the developing world, home is the site of many deaths of children under the age of 5 (1). These deaths are thought to be significantly influenced by acute respiratory infections (ARIs) (2). All across the world, ARIs are the main cause of morbidity and death for children under the age of 5 (3). Numerous pathogens are important contributors to childhood mortality due to respiratory illness. However, respiratory viruses are among these deadly agents during ARIs (4). Approximately 6.6 million children under the age of 5 die each year worldwide; 95% of these deaths occur in low-income nations, and ARIs account for one-third of all children’s deaths (5). In addition to that, ARIs cause 12 million morbidities and 1.3 million deaths in children under the age of 5 worldwide, with three-fourths of these deaths taking place in sub-Saharan Africa (SSA) (6). According to the World Health Organization (WHO) estimates, receiving timely and appropriate care could result in a 20% decrease in child deaths from ARIs (7). Mothers determine the kind and frequency of healthcare services that their children receive, so their proactive pursuit of care may reduce the risk of ARI-related morbidity and death (8). Approximately 85% of women in sub-Saharan African nations sought medical attention for illnesses that they had as children, where the highest and lowest prevalence were found in Gabon (75.0%) and Zambia (92.6%), respectively (9).

For ARIs in children under the age of 5 to be effectively treated, it is necessary to identify the condition of the child early, seek medical attention, and administer appropriate antibiotics as soon as possible (10). In a similar vein, only 40% of children in the SSA under the age of 5 who had ARI symptoms sought medical help (11). Mothers or other primary caregivers are essential in identifying the signs of an ARI in children under the age of 5 (12). The WHO is working on improving preventable child deaths and improving access and quality of care for newborns and children in primary healthcare services (13). Still, the goal of the integrated management of childhood illness (IMCI) program, which was introduced in low- and middle-income countries (LMICs), particularly in SSA, has not been met (14). Several studies on healthcare-seeking behavior for symptoms of ARIs among children under the age of 5 have been conducted in Bangladesh, Nigeria, east Africa, rural Kenya (15), Ghana, and sub-Saharan Africa (7, 11, 14, 16–19). Little is known by using cross-sectional studies, multivariate regression methods, bivariate analysis, multivariate logistic regression, and multilevel robust Poisson regression analysis. Furthermore, to the best of our knowledge, none of the previous researchers tried to predict mothers’ healthcare-seeking behavior for symptoms of ARIs among children aged 0–59 months using machine learning techniques. Therefore, this study aimed to predict healthcare-seeking behaviors of mothers with ARIs and their determinants among under-five children in SSA using different machine learning algorithms.

In summary, this study aimed to answer the following two main questions:

RQ1: Which determinants are the most significant for a mother’s healthcare-seeking behavior as a symptom of ARI?

RQ2: How can machine learning models help effectively predict a mother’s healthcare-seeking behavior?

## Materials and methods

### Study setting

This study was conducted in sub-Saharan African countries using the DHS dataset. Geographically, SSA is located south of the Sahara, which includes 50 internationally known countries. SSA is regionally classified into West Africa, Southern Africa, East Africa, and Central Africa.

### Data source

The data source in this study was the Measure DHS program, which was accessed via http://www.dhsprogram.com (20) after requesting and submitting the project title and the justification of the study. The DHS data are a nationally representative household survey collected periodically in various countries. For this study, we used the Kids Record dataset (KR file). Among the 50 SSA countries, 32 SSA countries’ DHS datasets were eligible for analysis. For the rest of the SSA countries, we did not get the DHS datasets.

### Population and eligibility criteria

All under-five children in SSA countries aged 0 to 59 months were considered the source population, while all under-five children who were in the selected enumeration areas (EAs) at the time of DHS data collection were the study populations and included in this study.

### Sample size determination and sampling technique

We used a weighted sample of 16,832 children aged 0–59 months across 32 sub-Saharan African countries using the recent DHS dataset. A two-stage stratified cluster sampling technique was used to select study participants (21). In the first step, a stratified sample of EAs was selected at random; in the second stage, households were selected using systematic random sampling in the selected EAs (21). In each selected household, mothers were interviewed using an individual questionnaire.

### Study variables and measurements

The target (outcome variable) in this study was mothers’ healthcare-seeking behavior status for children under the age of 5, which was measured by yes or no. Mothers who sought medical attention for ARI symptoms were classified as “yes,” while those who chose not to seek care were classified as “no.” The independent determinants of mothers’ healthcare-seeking behavior were extracted based on the literature review. Factors such as sociodemographic, socioeconomic, and community-level features are thought to be determinants of mothers’ healthcare-seeking behavior for ARIs among under-five children. The independent variables such as stunting, underweight, and wasting status, having diarrhea, birth size, marital status, sex child, region, and maternal occupation.

#### Operational definitions

##### Media exposure

Mothers seeking health information through media exposure, such as radio, television, and social media, get better awareness and knowledge to seek treatment for ARIs (22).

##### Stunting

Children who have stunted growth and development, often resulting from inadequate nutrition, infection, or stimulation (23).

##### Machine learning

“Machine learning is a branch of computer science that has the potential to transform epidemiologic sciences. Amid a growing focus on “Big Data,” it offers epidemiologists new tools to tackle problems for which classical methods are not well-suited” (24).

### Data analysis procedure

**Figure 1:**
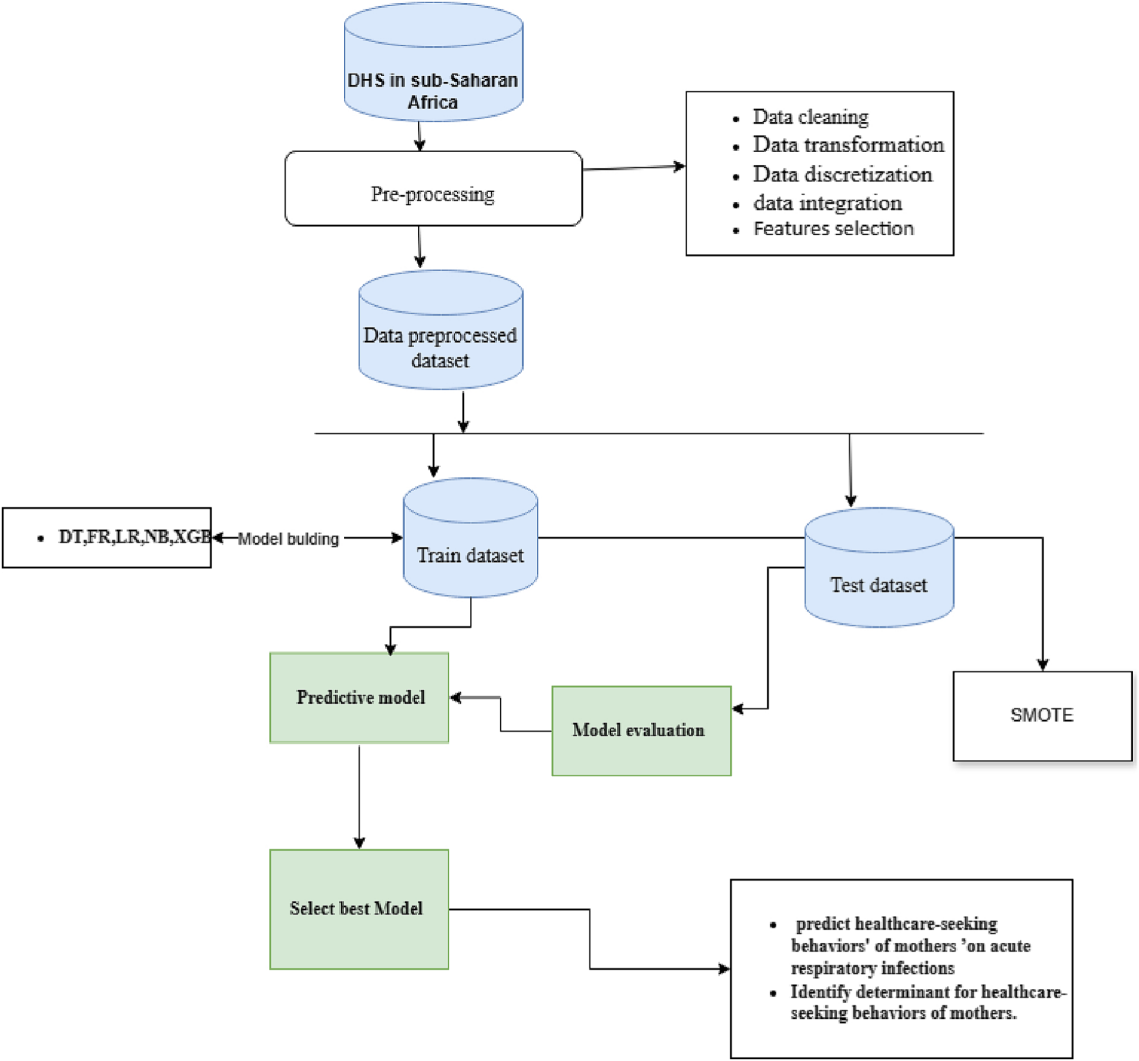
Flowchart of predictive healthcare-seeking behavior.

In this study, we utilized major data pre-processing techniques such as data cleaning, data transformation, data integration, and data discretization. Data processing and analysis were carried out using Python version 3.9, utilizing key packages such as Pandas, Scikit-Learn, Imblearn, Matplotlib, and Sklearn. In this study, key packages such as Pandas were used to import datasets, clean datasets, visualize datasets, and create aggregate datasets (25). In addition, the Scikit-Learn package is used for model selection, classification, prediction, and dimensionality reduction (26). The Imblearn package was also used for balancing datasets, which are biased toward some classes (27), while the Matplotlib and Skelearn packages were used for data visualization, which reduced massive data to tiny graphs for simple understanding (28). Data processing is a machine learning technique that transforms raw data into an understandable format (29). An understandable format entails making the data easily interpretable and suitable for analysis and modeling, thereby making it easier to work with, and tailoring it to meet specific requirements of a given task (29). After that, the pre-processed dataset was divided into two training and test datasets. To train the dataset, we used machine learning models such as random forests (RF), XG-Boost, decision trees, Naïve Bayes, and logistic regression algorithms, and the test dataset was used for performance evaluation for the proposed machine learning model. In this study, we developed a predictive model that determines both healthcare-seeking behavior and identifies determinants, as shown in Figure 1.

### Data pre-processing

Real-world data involve incredibly large numbers as well as noise, inconsistency, and missing data (30). To overcome these difficulties and make the data robust for classification, prediction, and forecasting, it is pre-processed (31).

#### Data cleaning

In this study, we used data pre-processing to reduce missing values, noise, inconsistency, and outliers. Approximately 2,245 (13%) of the 10 features in the dataset had missing values. To handle missing values in this study, we used mean and mode imputing methodologies to address these missing values for the continuous and categorical variables, respectively.

#### Outlier removing

We found outliers in the continuous nature of the data using visualization and statistical outlier detection methods, such as the box plot and the interquartile range (IQR), respectively. Subsequently, we removed these outliers.

#### Features selection

Second, each record in the dataset contains many features; therefore, feature selection is important. Bulk features are unsuitable for model building (32); therefore, feature selection is essential since unnecessary features during model training cause us to degrade the model’s overall accuracy, increase its complexity, limit its capacity to be generalized, and bias the model. In this study, we used wrapper feature selection-based proposed machine learning algorithms and SHAP values to select relevant features for model building.

#### Data transformation

On the other hand, data transformation entails transforming the data into a format that is appropriate for analysis; this may entail changing the data types, scaling, normalizing, or renaming (33). In this study, we employed one-hot-encoding techniques implemented using Python to convert categorical variables to dummy variables, with each category represented as a separate variable coded as 0 indicates mothers who have no health care-seeking behavior on ARI, and 1 indicates mothers who have health care-seeking behavior on ARI.

#### Data discretization and integration

To make the data easier to grasp and analyze, we did data discretization, where we converted continuous variables into discrete features according to DHS guidelines to minimize outlier influence and reduce noise. In this study, we integrated 32 countries’ datasets into a single dataset.

#### Class balancing

**Figure 2:**
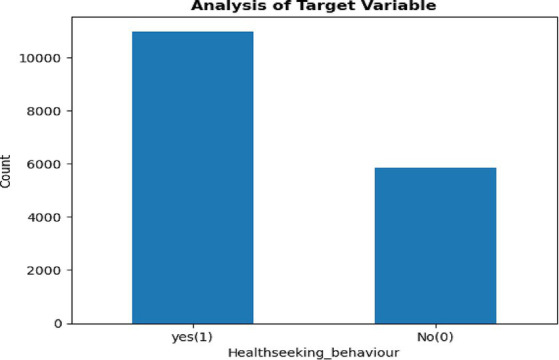
Prevalence of healthcare-seeking behavior status.

To avoid machine learning models biased toward the majority class, the training data were balanced using a synthetic minority oversampling technique (34). In Figure 2 show 10978 (65%) mothers who have health-seeking behavior on ARI whereas 5854 (35%) mothers who have no health seeking behavior on ARI. In this study, we used the adaptive synthetic sampling approach (ADSYN) oversampling by creating synthetic examples (new observations) that resemble the minority class by interpolating between minority class samples in the feature space rather than creating exact copies of existing examples.

### Machine learning classifiers

The advantage of machine learning is that it increases various subspecialties’ potential roles in health service delivery for children and adolescents. Machine learning is critical to predicting children’s and adolescents’ suicide risk accurately and within a manageable time frame (35). We proposed to use the machine learning method to predict a mother’s health when looking for signs of ARIs in children under the age of 5 and to provide insight for the government, policymakers, and governmental and non-governmental funders. The proposed RF, XG-Boost, decision trees, Naïve Bayes, and logistic regression algorithms were used to identify the determinants and build a predictive model. To efficiently handle massive volumes of survey data, we utilized a completely new integrated supervised machine learning algorithm created specifically for this research. The decision tree is one of the most popular models for predicting outcomes. Decision trees are highly accurate, stable, and easily interpretable predictive models that find the most important determinants. Because the tools can resolve issues with data fitting, they are also useful for fitting non-linear relationships (36).

Second, in the study, we proposed an RF, a combination of multiple decision trees that are used by the machine learning algorithm to generate predictions for classification and regression problems. RF has many advantages over traditional algorithms (37). RF overcomes these limitations of decision trees using an ensemble of decision trees (38).

#### Evaluation criteria

In this study, the performance of predictive models was evaluated by testing a dataset. We divided the data into training and test sets, with 13,465 samples (80%) allocated to the training set and 3,367 samples (20%) to the test set. Then, the performance of the trained models was evaluated using the test set based on the criteria of accuracy score, ROC curve, precision (P), recall (R), and F-measure as follows: The confusion matrix is a matrix of N * N, where N is the number of predicted classes, and it displays the number of correct and incorrect predictions made by the classification model relative to the target value shown in Table 1.

**Table 1:** Confusion matrix is a matrix.

N = Number of instances		Confirmed by observation	
Predicted by test		Yes	No
	Yes	TP (yes)	FP (Type 1 error)
	No	FN (Type 2 error)	TN (no)

Precision = (TP) / (TP + FP). Recall = (TP) / (TP + FN). F – Measure = (2 * Precision * Recall) / (Precision + Recall). Accuracy = ((TP + TN)/ (TP + TN + FP + FN)) × 100.

The models that are accurately classified as positive and predicted to be positive are referred to as true positives (TP). True negatives (TN) indicate that the model predicts the negative class as negative. A model that predicts the positive class incorrectly is referred to as a false positive (FP). False negatives (FN) are samples that are mistakenly labeled as belonging to a different class when in fact they are not (39). In addition, the receiver operating characteristics (ROC) curve filters the range of threshold values for decision-making and offers a thorough evaluation of a model’s accuracy (40).

## Results

### Sociodemographic and economic characteristics of participants

A weighted sample of 16,832 under-five children aged 0 to 59 months was included in this study. The majority (7,944, or 47.195%) of the mothers involved in this study were between the ages of 25 and 34. In this age range, 5,131 (30.48%) of mothers had followed healthcare-seeking behavior for ARIs among under-five children, and 7,652 (45.4%) of mothers with media exposure had healthcare-seeking behavior. The majority of the children included in this study, 12,398 (73.6%), lived in rural areas.

In approximately 4,573 (27%) of children who live in rural areas, the mother does not have the necessary healthcare-seeking behavior, which is significantly higher than the rate among urban children (1281) (7.6%). Of mothers who had media exposure, 7,652 (45.4%) had healthcare-seeking behavior compared to those who had not had media exposure, and 3,326 (19.7%) had healthcare-seeking behavior. Approximately 6,848 (40.6%) of children’s mothers who lived far from health facilities had no healthcare-seeking behavior, which is significantly higher than the rate among children who lived near health facilities. A total of 6,754 (40.12%) mothers had necessary healthcare-seeking behavior. The results of this study revealed that there is a low healthcare-seeking rate seen in South Africa (506; 3%) and Central Africa (2,360; 14.02%), as shown in Table 2.

**Table 2:** Description of sociodemographic and economic characteristics of participants.

Variable	Category	Frequency (%)	Healthcare-seeking behavior	
			Yes (%)	No (%)
Media_Exposure	No	5,941 (64.7%)	3,326 (19.76%)	2,615 (15.53%)
	Yes	10,891 (35.3%)	7,652 (45.4%)	3,239 (19.24%)
Distance health facility	Big problem	6,848 (40.68%)	2,624 (15.58%)	4,224 (25.095%)
	Not big problem	9,984 (59.32%)	6,754 (40.12%)	3,230 (19.18%)
Mother age	15–24 years	5,218 (31%)	3,484 (20.698%)	1734 (10.30%)
	25–34 years	7,944 (47.195%)	5,131 (30.48%)	2,813 (16.71%)
	35–49 years	3,670 (21.80%)	2,363 (14.03%)	1,307 (7.76%)
Place delivery	Home	6,145 (37%)	3,486 (21%)	2,659 (15.5%)
	Health facility	10,687 (63%)	7,492 (44.5%)	3,195 (19%)
Residence	Urban	4,434 (26.34%)	3,153 (18.73%)	1,281 (7.61%)
	Rural	12,398 (73.65%)	7,825 (46.48%)	4,573 (27.168%)
Wealth index	poorest	4,963 (29.48%)	2,964 (17.60%)	1999 (11.876%)
	Poorer	3,793 (22.53%)	2,451 (14.56%)	1,342 (7.972%)
	Middle	3,198 (18.99%)	2093 (12.43%)	1,105 (6.56%)
	Rich	2,714 (16.12%)	1839 (10.925%)	875 (5.198%)
	Richer	2,164 (12.85%)	1,631 (9.689%)	533 (3.166%)
Sub-Saharan Africa region	East Africa	7,061 (41.94%)	4,841 (28.76%)	2,220 (13.18%)
	Central Africa	4,177 (24.81%)	2,360 (14.02%)	1817 (10.79%)
	South Africa	703 (4.176%)	506 (3%)	197 (1.1%)
	West Africa	4,891 (29.05%)	3,271 (19.433%)	1,620 (9.62%)
ANC visit	Yes	15,671 (93.1%)	10,466 (62%)	5,205 (31%)
	No	1,161 (6.9%)	512 (3%)	649 (4%)
Maternal education	No education	6,023 (35.6%)	3,453 (21%)	2,570 (15.26%)
	Primary education	6,858 (41%)	4,649 (28%)	2,209 (13.12%)
	Secondary education	3,604 (21.4%)	2,594 (15.59%)	1,010 (6%)
	Higher education	347 (2%)	282 (2%)	65 (0.3%)
Stunting status	Normal	6,614 (39.3%)	4,308 (25.5%)	2,306 (14%)
	Moderate	1910 (11.3%)	1,200 (7%)	710 (4%)
	Severe	8,308 (49.4%)	5,470 (32.5%)	2,838 (17%)

(DHS, *n* = 16,832).

### Importance feature selection

**Figure 3:**
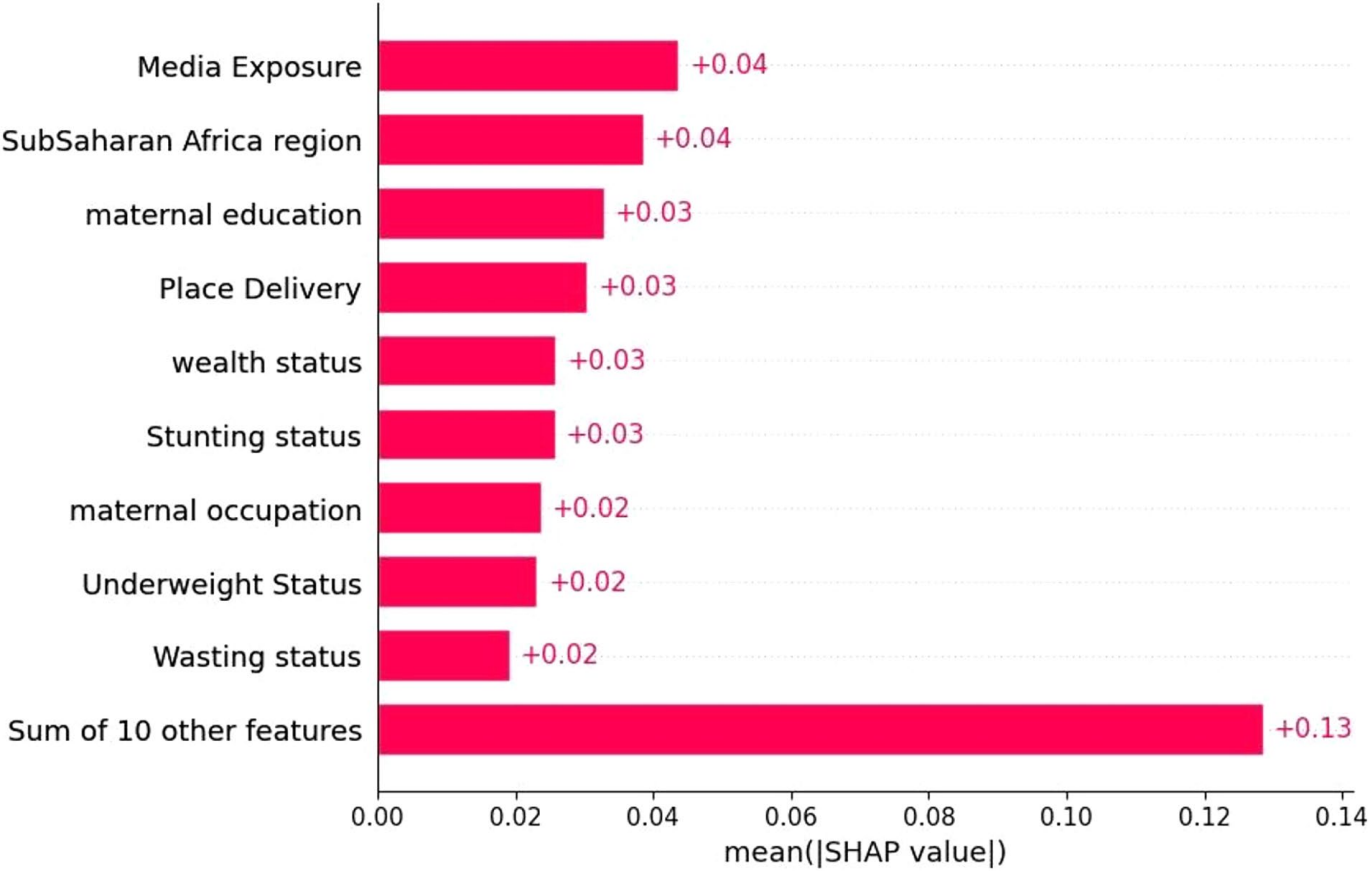
Important features with SHAP values.

**Figure 4:**
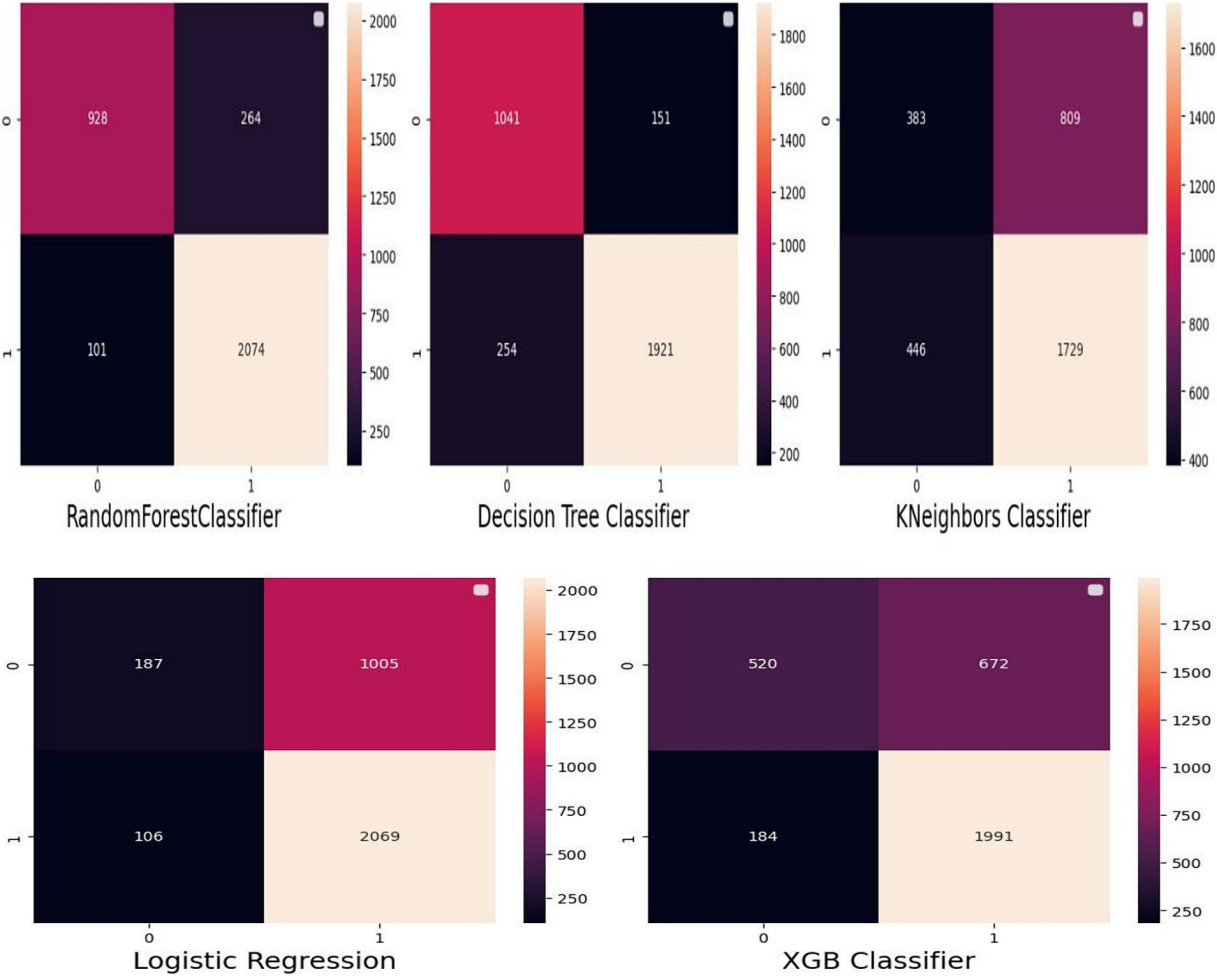
Confusion matrix.

In this study, before feature selection, the dataset comprised 16,832 rows and 121 columns. Including unnecessary features during model training can degrade the model’s overall accuracy, increase its complexity, limit its capacity to be generalized, and introduce bias (41). The importance of feature selection was to reduce the cost of learning by reducing the number of features. In this study, we deployed wrapper methods with SHAP values. The wrapper algorithm infers features’ relevance using the estimate of their importance from the best model. In this study, we selected important features based on RF, which were used to narrow down the set of potential features shown in Figure 3 and the SHAP values shown in Figure 4. Therefore, we used the already processed DHS in the sub-Saharan African clean dataset (16,832 rows and 19 columns) for our analysis.

### Class balancing

To make balanced data, we used the ADSYN oversampling technique to generate additional synthetic observations from the minority category to balance the unbalanced distribution of the outcome variable. Before applying adaptive synthetic sampling approach the Dependant variables observations was 10878 (65%), and 5854 (35%). After applying the ADSYN approach the overall distribution was changed to give an equal symmetric distribution in each class for both categories. After applying the ADSYN approach the overall distribution was changed to give an equal symmetric distribution in each class for both categories. For building reliable predictive models, we obtained a balanced sample dependent variable.

### ML classifier results

**Figure 5:**
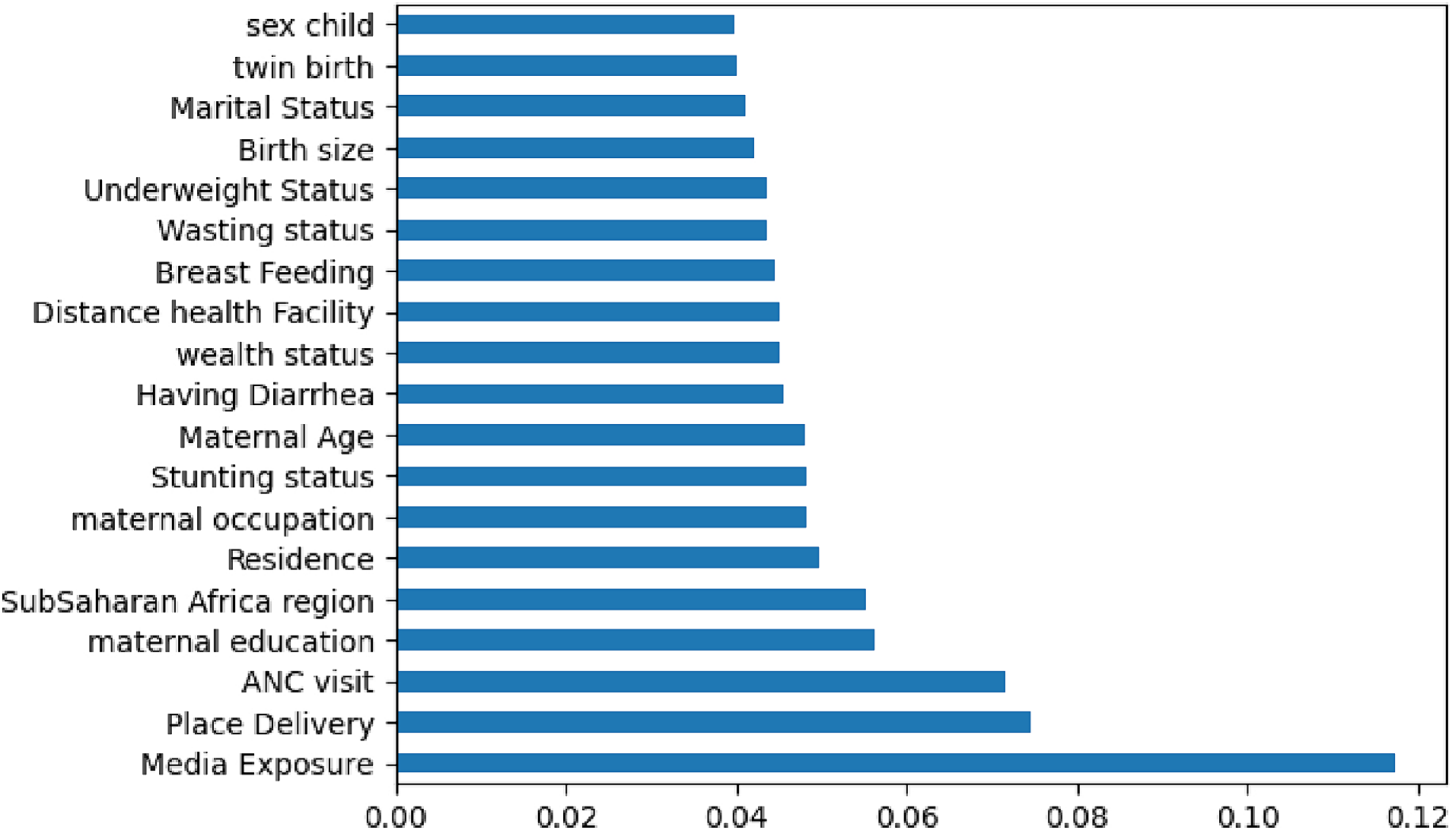
Important features selected by random forest.

**Figure 6:**
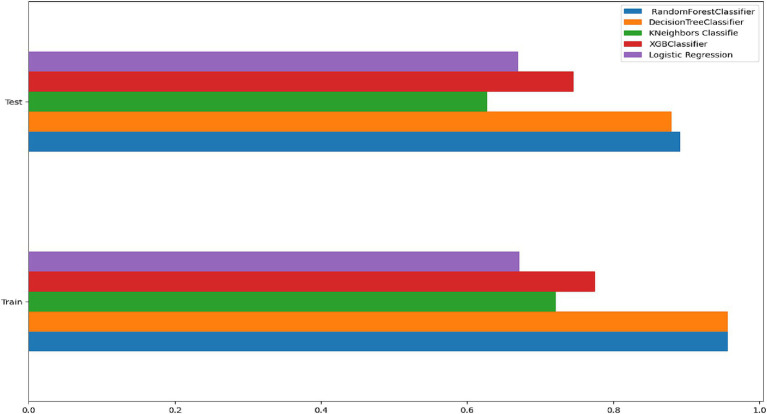
Compare the proposed machine learning model.

**Figure 7:**
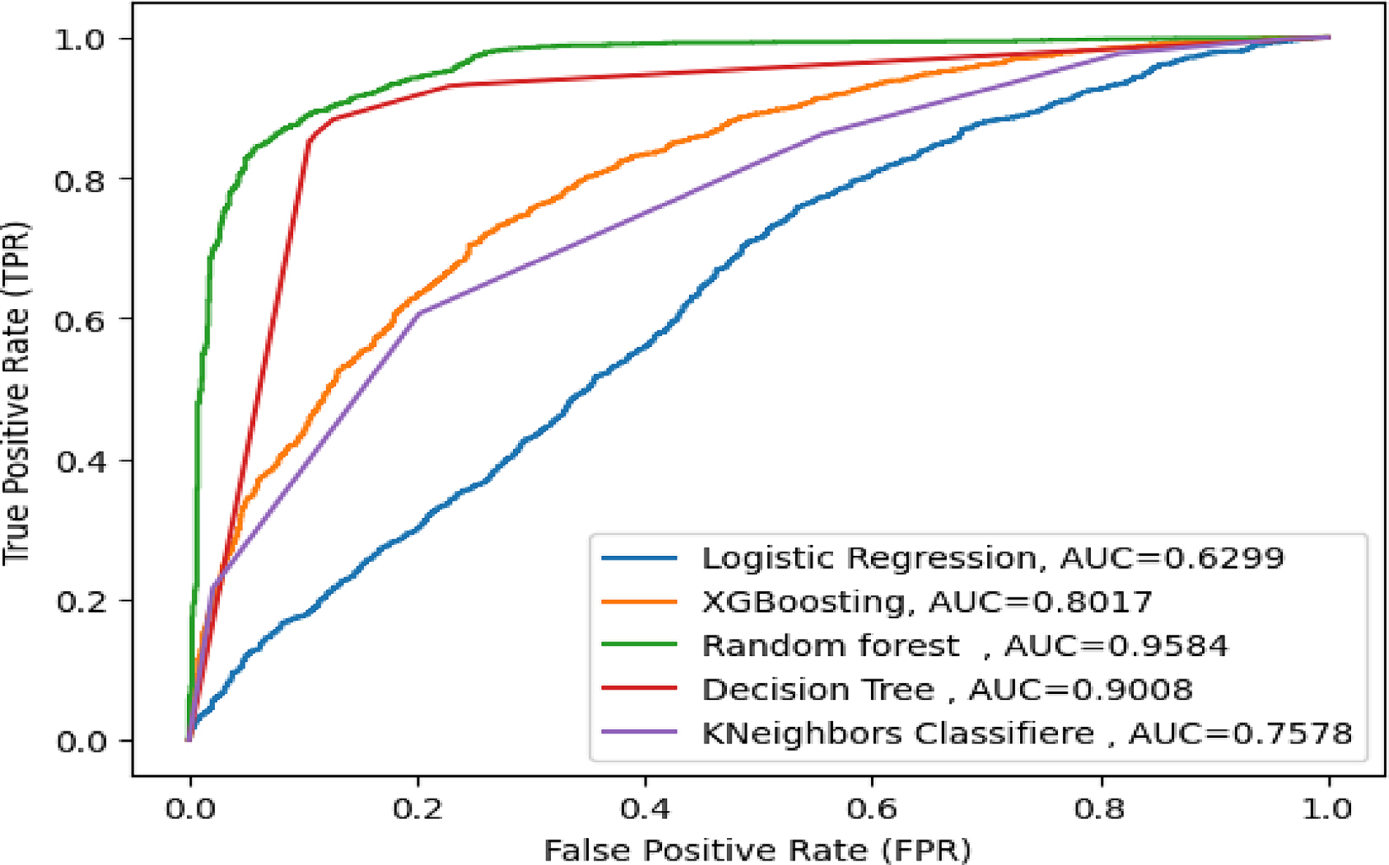
AUC for all proposed machine learning model.

In this study, the important determinants selected using the RF model and based on SHAP values are shown in Figures 3, 5. According to this study, the RF model had the highest predictive power and was able to identify factors associated with healthcare-seeking behavior for ARIs with 88.89% accuracy, 89.5% precision, 83% F-measure, 95.8% AUC ROC curve, and 77.6% recall. The average accuracy of all models was 77% or higher. Furthermore, the RF had high specificity (88.7%) and sensitivity (90.18%), with the lowest specificity observed with Naïve Bayes. The TP rate of RF was 77.85%, the FP rate was 22.14%, the TN rate was 95.3%, and the FN rate was 0.45% (Figure 4). Therefore, the RF model is good because the low FP rate and FN rate were compared to the TP rate and TN rate. In contrast, the Naïve Bayes model performance had a TP rate of 32.1%, a TN rate of 79.4%, an FP rate of 67%, and an FN rate of 20%; therefore, the Naïve Bayes model is highly mispredicted. According to SHAP values shown in Figure 3, the analysis indicated that the five most important variables were no media exposure, no maternal education, home delivery, poor wealth status, and region as key predictors of a mother’s healthcare-seeking behaviors as a symptom of ARI. In addition, the results of the proposed machine learning model are presented in Table 3 and Figures 6, 7 in terms of accuracy, precision, recall, AUCROC curve, and F-measure.

**Table 3:** Accuracy, precision, recall, and F-measure for the machine learning algorithms decision tree (DT), logistic regression (LR), extreme gradient boosting (XGB), and Naïve Bayes.

	Predictive model				
Evaluation matrix	Random forest	Logistic regression	Decision tree	XGBoost	Naïve Bayes
Metrics	%	%	%	%	%
Accuracy	88.89	67.15	87.97	74.52	66.41
Recall	95	95	88	91.6	84
Precision	88.5	68	92	74.6	70
F-score	91.7	78	90.4	74.5	76
AUC-score	95.8	63	90	80	75

## Discussion

This study used machine learning algorithms for predicting healthcare-seeking behaviors of mothers for ARIs and their determinants among under-five children in SSA that can be used for intervention.

This study showed that the RF model had the best predictive power and identified determinants for healthcare-seeking behavior with an accuracy of 88.89%, a precision of 89.5%, an F-measure of 83%, an AUC ROC curve of 95.8%, and a recall of 77.6% when compared with the proposed machine learning classifier models such as DT, XGB, Naïve Bayes, and logistic regression. Moreover, the RF model correctly predicted mothers’ healthcare-seeking behavior with a sensitivity of 90.18% and a specificity of 88.71%. Additionally, the predicted RF model had PPV and NPV of 77.85 and 95.35%, respectively. The predicted RF model incorrectly predicted 101 (9.82%) mothers who had healthcare-seeking behavior and 264 (11.29%) mothers who had no healthcare-seeking behavior. This revealed that the predicted ML model has very good accuracy and model performance, with minimum FP and FN rates.

The results of the RF model feature selection showed that the following factors were significant predictors of a mother’s healthcare-seeking behavior for symptoms of ARI: no media exposure, living in rural areas, not breastfeeding, poor wealth status, home delivery, no ANC visit, no maternal education, mothers’ age group of 35–49 years, and distance to health facilities were significant predictors for the absence of mothers’ healthcare-seeking behaviors for ARIs. On the other hand, factors such as undernourished children (stunting, underweight, and wasting status), having diarrhea, having a large birth size, being married, the child’s sex (male or female), region, and maternal occupation were significantly associated with good maternal healthcare-seeking behaviors for ARIs among under-five children.

This study showed that mothers with media exposure were more likely to have healthcare-seeking behavior compared with those who had no media exposure. Mothers of children who had media exposure, such as newspapers, radio, and television, played a critical role in gaining better awareness and knowledge to seek treatment for ARI. These findings are supported by similar findings in Ethiopia (16). Because of media exposure, mothers are more able to recognize the signs of respiratory infections, and as a result, they visit hospitals to seek care (11, 17). Similarly, distance to a health facility was one of the determinants of healthcare-seeking behavior for Muslims.

Mothers who were far from a health facility were less likely to have healthcare-seeking behaviors compared to those who were near the facility. Many studies in Uganda and southwest Ethiopia (17, 42, 43) supported this finding. This might be because mothers who were far from the health facility were less inclined to go for medical care. This is especially common in developing countries such as SSA because of high transportation costs and inadequate infrastructure (7, 43). Similarly, mothers who gave birth at a medical facility were more likely to seek medical attention than mothers who gave birth at home (11). Mothers may find it more challenging to give birth in medical facilities if there are inadequate facilities. Our study found that children who were delivered in hospitals had higher rates of total healthcare utilization and visits to hospitals for immunizations and postpartum care. If a mother has any symptoms of ARI while going to these services, she may bring her child for medical attention. Similar findings corroborate the conclusions (44).

Similarly, it has been demonstrated that mothers who are educated are more likely to have healthcare-seeking behavior for ARIs. This result is also consistent with research carried out in Ethiopia (14, 17, 45). Mothers who have completed primary school or higher are more likely to seek medical attention and remedies for their children, as well as to take better care of them (46). Compared to moms in rural regions, mothers in urban areas were more likely to seek medical attention at a health facility for ARIs for their children. This is verified by earlier research findings (47). Mothers who live in urban areas are more likely to be exposed to the media and have access to healthcare facilities than those who reside in rural areas.

Similarly, in terms of the Wealth Index, families with higher wealth status were linked to a greater propensity to seek medical attention from a facility than those with lower wealth status (47, 48). This study also revealed one important factor in the decision to seek care in a medical facility was income, and women with money would bring their children to the hospital when they displayed signs of ARI.

A machine learning model is important for evidence-based decision-making, and a prediction application is an application that can early predict healthcare-seeking behaviors based on the symptoms of ARIS only by entering important features as a result of measurement. Additionally, machine learning models can find hidden patterns in data, which makes it possible to extrapolate such patterns to new, previously undetected data.

## Conclusion and recommendation

Machine learning approaches can be used to classify certain hidden information that is indiscernible using conventional statistical tools. The findings of the last experiment showed that the RF model was the most accurate at evaluating risk factors and predicting healthcare-seeking behaviors related to ARI. The model of the RF technique selected the more important determinants, such as no media exposure, living in rural areas, not breastfeeding, poor wealth status, home delivery, no ANC visit, no maternal education, mothers’ age group of 35–49 years, and distance to health facilities, as significant predictors for the absence of mothers’ healthcare-seeking behaviors for ARIs. On the other hand, undernourished children, such as stunting, underweight, and wasting status, diarrhea, birth size, married women, being a male or female sex child, region, and maternal occupation, were significantly associated with good maternal healthcare-seeking behaviors for ARIs among under-five children.

Therefore, policymakers should consider the findings of the research and develop a plan for boosting healthcare-seeking behaviors in sub-Saharan nations based on the variables that have been found to be significant. Despite the intriguing outcome, more studies need to be conducted using different kinds of approaches with different parameters. It is also advised that mothers get media exposure interventions regarding the importance of seeking proper medical attention at health facilities for symptoms of ARIs.

## Strength and limitations

This study more precisely assessed the important factors and tried to forecast mothers’ healthcare-seeking behaviors based on ARI symptoms. Additionally, the study used the most recent DHS dataset of sub-Saharan African countries, which includes nearly all vulnerable demographic risk groups. Because DHS data collection is self-reported, there may have been some information biases added, and the limitations of DHS do not include behavioral factors such as attitude and knowledge factors, and we did not include important variables such as clinically related variables, which limits this study.

In this study, the implementation challenges of the proposed machine learning model take a tremendous amount of time to build and test, as well as to predict the best output.

## Data availability statement

The original contributions presented in the study are included in the article/supplementary materials, further inquiries can be directed to the corresponding author.

## Ethics statement

Ethical approval was not required for the studies involving humans because datasets used in this analysis are publicly available in the DHS Program repository. The studies were conducted in accordance with the local legislation and institutional requirements. Written informed consent for participation was not required from the participants or the participants’ legal guardians/next of kin in accordance with the national legislation and institutional requirements because we were granted permission to access the data via the measure DHS program online request form on the website (https://dhsprogram.com/).

## Author contributions

TY: Data curation, Methodology, Software, Writing – original draft, Writing – review & editing. MA: Conceptualization, Data curation, Writing – review & editing. ND: Investigation, Supervision, Validation, Writing – original draft. SW: Methodology, Software, Supervision, Writing – review & editing. BF: Data curation, Formal analysis, Investigation, Writing – original draft. GY: Conceptualization, Data curation, Writing – review & editing. TT: Formal analysis, Investigation, Methodology, Writing – review & editing.

## References

[1] Kouao A N’datchoh E Yoboue V Silue S Attoh H Coulibaly M. Exposure to indoor and outdoor air pollution among children under five years old in urban area. Glob J Environ Sci Manag. (2019) 5:191–202. doi: 10.22034gjesm.2019.02.05

[2] Merera AM. Determinants of acute respiratory infection among under-five children in rural Ethiopia. BMC Infect Dis. (2021) 21:1–12. doi: 10.1186/s12879-021-06864-4 34847859 PMC8631694

[3] Safiri S Mahmoodpoor A Kolahi A-A Nejadghaderi SA Sullman MJ Mansournia MA. Global burden of lower respiratory infections during the last three decades. Front Public Health. (2023) 10:1028525. doi: 10.3389/fpubh.2022.1028525, PMID: 36699876 PMC9869262

[4] Li Y Wang X Blau DM Caballero MT Feikin DR Gill CJ. Global, regional, and national disease burden estimates of acute lower respiratory infections due to respiratory syncytial virus in children younger than 5 years in 2019: a systematic analysis. Lancet. (2022) 399:2047–64. doi: 10.1016/S0140-6736(22)00478-0, PMID: 35598608 PMC7613574

[5] Tazinya AA Halle-Ekane GE Mbuagbaw LT Abanda M Atashili J Obama MT. Risk factors for acute respiratory infections in children under five years attending the Bamenda regional Hospital in Cameroon. BMC Pulm Med. (2018) 18:1–8. doi: 10.1186/s12890-018-0579-7 29338717 PMC5771025

[6] Tesema GA Worku MG Alamneh TS Teshale AB Yeshaw Y Alem AZ. Understanding the rural–urban disparity in acute respiratory infection symptoms among under-five children in sub-Saharan Africa: a multivariate decomposition analysis. BMC Public Health. (2022) 22:1–12. doi: 10.1186/s12889-022-14421-0 36324089 PMC9632025

[7] Ndungu EW Okwara FN Oyore JP. Cross sectional survey of care seeking for acute respiratory illness in children under 5 years in rural Kenya. Am J Pediatr. (2018) 4:69–79. doi: 10.11648/j.ajp.20180403.15

[8] Sultana M Sarker AR Sheikh N Akram R Ali N Mahumud RA. Prevalence, determinants and health care-seeking behavior of childhood acute respiratory tract infections in Bangladesh. PLoS One. (2019) 14:e0210433. doi: 10.1371/journal.pone.0210433, PMID: 30629689 PMC6328134

[9] Ahinkorah BO Budu E Seidu A-A Agbaglo E Adu C Ameyaw EK. Barriers to healthcare access and healthcare seeking for childhood illnesses among childbearing women in sub-Saharan Africa: a multilevel modelling of demographic and health surveys. PLoS One. (2021) 16:e0244395. doi: 10.1371/journal.pone.0244395, PMID: 33556057 PMC7870045

[10] Ujunwa F Ezeonu C. Risk factors for acute respiratory tract infections in under-five children in Enugu Southeast Nigeria. Ann Med Health Sci Res. (2014) 4:95–9. doi: 10.4103/2141-9248.126610, PMID: 24669339 PMC3952306

[11] Chilot D Shitu K Gela YY Getnet M Mulat B Diress M. Factors associated with healthcare-seeking behavior for symptomatic acute respiratory infection among children in East Africa: a cross-sectional study. BMC Pediatr. (2022) 22:662. doi: 10.1186/s12887-022-03680-w, PMID: 36380283 PMC9664707

[12] Al Fidah MF Nabin AA Efa SS. Factors associated with acute respiratory infection and healthcare-seeking behaviour among primary caregivers in Bangladesh: a study based on MICS 2019. BMJ Public Health. (2024) 2. doi: 10.1136/bmjph-2023-000576 PMC11812817 40018135

[13] Titaley CR Que BJ de Lima FV Angkejaya OW de Lima FV Maelissa MM. Health care–seeking behavior of children with acute respiratory infections symptoms: analysis of the 2012 and 2017 Indonesia demographic and health surveys. Asia Pac J Public Health. (2020) 32:310–9. doi: 10.1177/1010539520944716, PMID: 32729324

[14] Tesema GA Seifu BL. Factors associated with mother's healthcare-seeking behavior for symptoms of acute respiratory infection in under-five children in sub-Saharan Africa: a multilevel robust Poisson regression modelling. BMC Health Serv Res. (2023) 23:1061. doi: 10.1186/s12913-023-10065-x, PMID: 37794438 PMC10552283

[15] Khasanah U Efendi F Has EMM Adnani QES Ramadhan K Arna YD. Healthcare-seeking behavior for children aged 0–59 months: evidence from 2002–2017 Indonesia demographic and health surveys. PLoS One. (2023) 18:e0281543. doi: 10.1371/journal.pone.0281543, PMID: 36758015 PMC9910639

[16] Badolo H Bado AR Hien H Méda N Susuman AS. Factors associated with mothers’ health care-seeking behaviours for childhood fever in Burkina Faso: findings from repeated cross-sectional household surveys. Glob Health Res Policy. (2022) 7:37. doi: 10.1186/s41256-022-00270-2, PMID: 36266714 PMC9585735

[17] Yaya S Odusina EK Adjei NK. Health care seeking behaviour for children with acute childhood illnesses and its relating factors in sub-Saharan Africa: evidence from 24 countries. Trop Med Health. (2021) 49:1–8. doi: 10.1186/s41182-021-00385-1 34906263 PMC8670049

[18] Adeoti IG Cavallaro FL. Determinants of care-seeking behaviour for fever, acute respiratory infection and diarrhoea among children under five in Nigeria. PLoS One. (2022) 17:e0273901. doi: 10.1371/journal.pone.0273901, PMID: 36107948 PMC9477346

[19] Mukandoli E. Health seeking behaviors of parents/caretakers of children with severe respiratory infections in a selected referral hospital in. Rwanda: University of Rwanda (2017).

[20] Terefe B Jembere MM. Discrimination against HIV/AIDS patients and associated factors among women in east African countries: using the most recent DHS data (2015–2022). J Health Popul Nutr. (2024) 43:3. doi: 10.1186/s41043-023-00491-2, PMID: 38167573 PMC10759423

[21] Rajput D Wang W-J Chen C-C. Evaluation of a decided sample size in machine learning applications. BMC Bioinformatics. (2023) 24:48. doi: 10.1186/s12859-023-05156-9, PMID: 36788550 PMC9926644

[22] Chonmaitree T Trujillo R Jennings K Alvarez-Fernandez P Patel JA Loeffelholz MJ. Acute otitis media and other complications of viral respiratory infection. Pediatrics. (2016) 137:e20153555. doi: 10.1542/peds.2015-3555, PMID: 27020793 PMC4811317

[23] Agrafiotis M Siempos II Falagas ME. Infections related to airway stenting: a systematic review. Respiration. (2009) 78:69–74. doi: 10.1159/000213244, PMID: 19365108

[24] Bi Q Goodman KE Kaminsky J Lessler J. What is machine learning? A primer for the epidemiologist. Am J Epidemiol. (2019) 188:2222–39. doi: 10.1093/aje/kwz189, PMID: 31509183

[25] Molin S. Hands-On Data Analysis with Pandas: Efficiently perform data collection, wrangling, analysis, and visualization using Python. Birmingham - Mumbai: Packt Publishing Ltd. (2019).

[26] Kabir MF Chen T Ludwig SA. A performance analysis of dimensionality reduction algorithms in machine learning models for cancer prediction. Healthc Anal. (2023) 3:100125. doi: 10.1016/j.health.2022.100125

[27] Hamama M. A study imbalance handling by various data sampling methods in binary classification. arXiv preprint arXiv. (2021):210510959. doi: 10.48550/arXiv.2105.10959

[28] Sial AH Rashdi SYS Khan AH. Comparative analysis of data visualization libraries Matplotlib and Seaborn in Python. Int J. (2021) 10:45. doi: 10.30534/ijatcse/2021/391012021

[29] Kang M Tian J. Machine learning: data pre-processing. Prognostics and Health Management of Electronics. eds. Michael G Pecht MK (USA: John Wiley et Sons) (2018). 111–30.

[30] Emmanuel T Maupong T Mpoeleng D Semong T Mphago B Tabona O. A survey on missing data in machine learning. J Big Data. (2021) 8:1–37. doi: 10.1186/s40537-021-00516-9 34722113 PMC8549433

[31] Karmaker A Kwek S. An iterative refinement approach for data cleaning. Intell Data Anal. (2007) 11:547–60. doi: 10.3233/IDA-2007-11507

[32] Marcos-Zambrano LJ Karaduzovic-Hadziabdic K Loncar Turukalo T Przymus P Trajkovik V Aasmets O. Applications of machine learning in human microbiome studies: a review on feature selection, biomarker identification, disease prediction and treatment. Front Microbiol. (2021) 12:634511. doi: 10.3389/fmicb.2021.634511, PMID: 33737920 PMC7962872

[33] Kebede SD Sebastian Y Yeneneh A Chanie AF Melaku MS Walle AD. Prediction of contraceptive discontinuation among reproductive-age women in Ethiopia using Ethiopian demographic and health survey 2016 dataset: a machine learning approach. BMC Med Inform Decis Mak. (2023) 23:1–17. doi: 10.1186/s12911-023-02102-w 36650511 PMC9843668

[34] Soltanzadeh P Hashemzadeh M. RCSMOTE: range-controlled synthetic minority over-sampling technique for handling the class imbalance problem. Inf Sci. (2021) 542:92–111. doi: 10.1016/j.ins.2020.07.014

[35] Su C Aseltine R Doshi R Chen K Rogers SC Wang F. Machine learning for suicide risk prediction in children and adolescents with electronic health records. Transl Psychiatry. (2020) 10:413. doi: 10.1038/s41398-020-01100-0, PMID: 33243979 PMC7693189

[36] Li X Yi S Cundy AB Chen W. Sustainable decision-making for contaminated site risk management: a decision tree model using machine learning algorithms. J Clean Prod. (2022) 371:133612. doi: 10.1016/j.jclepro.2022.133612

[37] Feng W Ma C Zhao G Zhang R, editors. Fsrf: an improved random forest for classification. 2020 IEEE International Conference on Advances in Electrical Engineering and Computer Applications (AEECA). (2020). China: IEEE.

[38] Sun Z Wang G Li P Wang H Zhang M Liang X. An improved random forest based on the classification accuracy and correlation measurement of decision trees. Expert Syst Appl. (2024) 237:121549. doi: 10.1016/j.eswa.2023.121549

[39] Beauxis-Aussalet E Hardman L. Simplifying the Visualization of Confusion Matrix. Amsterdam: BNAIC. (2014).

[40] Nahm FS. Receiver operating characteristic curve: overview and practical use for clinicians. Korean J Anesthesiol. (2022) 75:25–36. doi: 10.4097/kja.21209, PMID: 35124947 PMC8831439

[41] Khalid S Khalil T Nasreen S, editors. A survey of feature selection and feature extraction techniques in machine learning. 2014 Science and Information Conference; (2014). UK: IEEE.

[42] Musoke D Boynton P Butler C Musoke MB. Health seeking behaviour and challenges in utilising health facilities in Wakiso district, Uganda. Afr Health Sci. (2014) 14:1046–55. doi: 10.4314/ahs.v14i4.36, PMID: 25834516 PMC4370086

[43] Begashaw B Tessema F Gesesew HA. Health care seeking behavior in Southwest Ethiopia. PLoS One. (2016) 11:e0161014. doi: 10.1371/journal.pone.0161014, PMID: 27626804 PMC5023186

[44] Gebrerufael GG Hagos BT. Prevalence and predictors of acute respiratory infection among children under-five years in Tigray regional state, northern Ethiopia: a cross sectional study. BMC Infect Dis. (2023) 23:743. doi: 10.1186/s12879-023-08701-2, PMID: 37904115 PMC10614314

[45] Simieneh MM Mengistu MY Gelagay AA Gebeyehu MT. Mothers’ health care seeking behavior and associated factors for common childhood illnesses, Northwest Ethiopia: community based cross-sectional study. BMC Health Serv Res. (2019) 19:1–7. doi: 10.1186/s12913-019-3897-4 30674309 PMC6343298

[46] Kiross GT Chojenta C Barker D Tiruye TY Loxton D. The effect of maternal education on infant mortality in Ethiopia: a systematic review and meta-analysis. PLoS One. (2019) 14:e0220076. doi: 10.1371/journal.pone.0220076 31356599 PMC6663004

[47] Ng’ambi W Mangal T Phillips A Colbourn T Nkhoma D Mfutso-Bengo J. A cross-sectional study on factors associated with health seeking behaviour of Malawians aged 15+ years in 2016. Malawi Med J. (2020) 32:205–12. doi: 10.4314/mmj.v32i4.5, PMID: 34457205 PMC8364791

[48] Danquah L Amegbor PM Ayele DG. Determinants of the type of health care sought for symptoms of acute respiratory infection in children: analysis of Ghana demographic and health surveys. BMC Pediatr. (2021) 21:1–14. doi: 10.1186/s12887-021-02990-9 34789184 PMC8597276

